# Supplementary material for: Identification of photoperiod-induced specific miRNAs in the adrenal glands of Sunite sheep (Ovis aries)
Source: Front Vet Sci. 2022 Jul 22;9:888207. doi: 10.3389/fvets.2022.888207 (PMC9354845; doi:10.3389/fvets.2022.888207)
Supplement: Supplementary file 1 [file Data_Sheet_1.ZIP › Supplementary file/miRDeep2 file.zip!/miRDeep2 file/Novel_Structure/SPLP42A2/Novel_512.pdf]

Provisional ID	:	NC_019466.2_19413
Score total	:	0.2
Score for star read(s)	:	-1.3
Score for read counts	:	-1.8
Score for mfe	:	1.8
Score for randfold	:	1.6
Score for cons. seed	:	
Total read count	:	8
Mature read count	:	8
Loop read count	:	0
Star read count	:	0

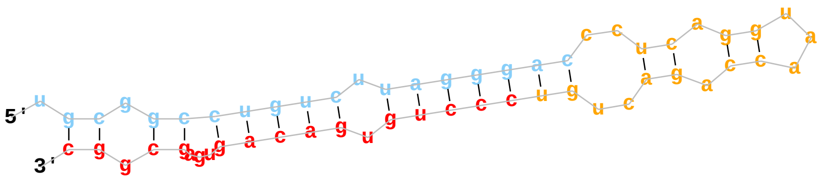

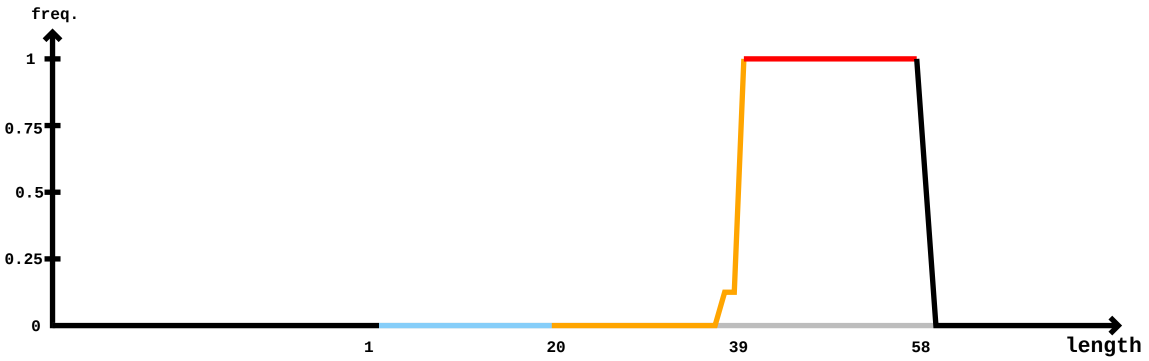

Star Mature

5'- gguuugggauaagacuuagugguuugcagaacugcggccugucuuagggacccucagguaaccagacugucccugugacagugagcggcuguaaacauccucgacugga -3'	exp			
((((.(((((...........((((((((..((((...(((((.(((((((..((.((...)).))..))))))).)))))...))))))))))))))))).))))...		reads	mm	sample
....gucccugugacagugagcggc.....................		1	0	seq
....cccugugacagugagcggc.....................		7	0	seq
